# “Too Much to Ask, Too Much to Handle”: Women’s Coping in Times of Zika

**DOI:** 10.3390/ijerph17124613

**Published:** 2020-06-26

**Authors:** Ana Rosa Linde Arias, Elisa Tristan-Cheever, Grace Furtado, Eduardo Siqueira

**Affiliations:** 1 Mauricio Gastón Institute for Latino Community Development and Public Policy, University of Massachusetts Boston, Boston, MA 02125-3393, USA; Grace.Furtado001@umb.edu; 2 Escola de Matemática Aplicada FGV EMAp, Fundação Getulio Vargas FGV, Rio de Janeiro, RJ 22250-900, Brazil; 3 Cambridge Health Alliance, Cambridge, MA 02139, USA; etristan-cheever@challiance.org; 4 School for the Environment, University of Massachusetts Boston, Boston, MA 02125-3393, USA; carlos.siqueira@umb.edu

**Keywords:** Zika, life impacts, psychosocial impacts, public health, epidemics, health protection measures, social determinants of health, cultural factors

## Abstract

Zika virus infection during pregnancy is a cause of congenital brain abnormalities. Its consequences for pregnancies have made governments and both national and international agencies issue advice and recommendations to women. This study was designed to understand the impacts of Zika on women who were less directly affected and less vulnerable to Zika. Women were recruited from various locations in Brazil, Puerto Rico, and the United States. Data were collected through semi-structured interviews and analyzed using thematic analysis. Women perceived that public health systems placed an unfair responsibility for preventing health complications from Zika onto women who had limited ability to do so. They also stated that the measures recommended to them were invasive, while creating the perception that women were the sole determinant of whether they contracted Zika. The results indicate that women with higher levels of education understood the limitations of the information, government actions, and medical care they received, which ended up producing higher levels of anguish and worry. Gender inequality and discrimination must be recognized and rendered visible in the public health emergency response. The social effects of the epidemic affected women more than had been thought before and at deeper emotional levels.

## 1. Introduction

In May 2015, the Pan American Health Organization issued an alert reporting the first confirmed Zika virus infection in Brazil. Subsequently, on 1 February 2016, the World Health Organization (WHO) declared that a massive Zika virus outbreak in the Americas was a Public Health Emergency of International Concern [1]. Today, while news media outlets are no longer concerned with Zika, and WHO recently downgraded the virus from a public health emergency to a “common threat”, more than 70 countries and U.S. territories continue to report active transmissions of Zika [2]. Without a doubt, concern should be raised not only regarding the likelihood of the virus persisting endlessly in current areas, but also about the danger of it spreading to new areas throughout the globe.

Generally, the Zika virus causes only a mild infection in humans, but it can produce severe neurological complications and adverse fetal outcomes [3]. During pregnancy, Zika virus infection can trigger congenital brain abnormalities [4]. Moreover, sexual transmission of Zika from both male and female partners can occur [5,6] and the virus can remain viable in semen for months [7]. The risk of birth defects resulting from Zika infection during early pregnancy makes the virus an especially significant health threat for pregnant women and, more generally, women of childbearing age.

These unique consequences of Zika for maternal and perinatal health call for the implementation of a broad spectrum of public health interventions. Governments, along with national and international agencies, have issued general advice and recommendations to safeguard women from infection [8]. More specifically, many countries officially advised women to avoid becoming pregnant in Zika-affected areas [9], whereas others stressed the importance of obtaining counseling and sufficient access to family planning resources [10]. Even more, by locating reproduction solely within women’s bodies, men’s sexualities and reproductive capacities were placed outside the purview of Zika epidemic responses.

Preventive measures also include recommendations such as avoiding locations likely to contain infected mosquitos, covering oneself completely with clothing, and applying repellents, among other measures [11,12]. Overall, there has been great concern with the biomedical aspects of disease transmission with regard to how transmission can be prevented through new vaccines or detection methods, and how to combat the proliferation of the vector of the virus. The public health response to the Zika outbreak has mostly focused on epidemiological surveillance and vector control.

Another important factor to consider is how the political contexts and history of a country influence the population reaction to an epidemic such as Zika. Many countries in the Americas where Zika was present had challenging legal, social, political and economic structures. For instance, Brazil, which had the greatest number of Zika cases, is a country with high levels of socioeconomic inequality, restrictive abortion legislation, and structural deficiencies in sanitation and health care. Most women and children affected by Zika lived in difficult conditions. In Brazil, there is evidence of a strong association between a higher prevalence of microcephaly and low socioeconomic level [13]. The prevalence of live births with microcephaly was higher among mothers who were non- white, had lower education levels, lived in the Northeast, and reported to be single or living with a steady partner. These inequalities in the impacts of Zika were also reported in other countries [14]. Puerto Rico, also greatly affected by the epidemic, had high levels of socioeconomic inequality, restrictive reproductive rights laws, weak governance, and a strong economic crisis.

Few studies have been conducted to address the threatening effects that the Zika epidemic has on women’s lives at a transnational level. Likewise, the ways in which women are psychologically and emotionally dealing with the threat of Zika outbreaks have yet to be documented. The psychosocial implications of the Zika epidemic are essential for a complete understanding of its long-term repercussions. To our knowledge, few studies discuss the impacts on women who are less directly affected and less vulnerable to Zika.

This study was designed to develop an initial understanding of the impacts of Zika on women of reproductive age who lived in areas with or without local transmission. Next, we describe and discuss the impacts of the Zika epidemic on women who either lived in areas where Zika infection was present or were indirectly affected by the epidemic. The impacts on women who were indirectly affected and less vulnerable to Zika were analyzed. Our hypothesis was that Zika impacted the daily lives of all women, directly or indirectly.

## 2. Methodology

This study adopted a qualitative research approach. Qualitative inquiry is used to explore and describe a poorly understood phenomenon that happens within a given context [15]. Qualitative interviews allow us to gather detailed information and afford opportunities to explore matters that are unique to the experiences of the interviewees in an in-depth manner, allowing insights into how different social processes of interest are experienced and perceived [16].

### 2.1. Data Collection

We conducted semi-structured interviews with 30 women who lived in various locations in Brazil, Puerto Rico, and the United States, and self-identified as Brazilian, Hispanic or American. They had different nationalities, socioeconomic positions, religions, ages, and cultural backgrounds. They resided in two countries and a U.S. territory that have different legal systems, public health policies, and socio-cultural contexts. We chose Brazil and the U.S. as sites for the study because all authors live in Massachusetts but have professional and personal connections in Brazil, the continental U.S., and Puerto Rico.

Participants were recruited by personal contacts of the research team and community associations through the snowball sampling technique. Therefore, several of the interviewees trusted the interviewer and provided sensitive and confidential information about their lives. Sampling continued until data saturation. Snowball sampling is widely applied in qualitive studies when samples with the target characteristics are not easily accessible. It allows access to “hard to reach” vulnerable populations who would otherwise be very difficult to recruit in large numbers [17]. We recruited women of reproductive age (18–45 years old) living or migrating from countries (such as Brazil, Venezuela, or Colombia) or U.S. states where the Zika virus was detected.

The interview guide was pilot tested with a couple of interviewees to test for reliability and validity. It included topics such as women’s personal and family life, perceptions and knowledge of Zika, views on reproductive health and rights related to the Zika syndrome. It was revised according to the feedback provided and allowed for participants to suggest new topics of interest. As an example, after the first pilot interviews, modifications were made on questions about women’s health literacy about Zika, since authors realized that participants responded mostly based on their knowledge rather than perceptions and personal experiences with Zika.

Additional in-depth interviews with six key female informants, who worked directly with women affected by the Zika virus, were also conducted to collect expert information on the impacts on such women. These key informants included: a female official of the Brazilian Ministry of Health in charge of providing assistance to mothers of children with microcephaly; a female psychologist who provided care to mothers in Rio de Janeiro, Brazil; a female pediatrician in Boston who worked with Latino communities; a female researcher in Puerto Rico who worked in Public Health; a female doctor in Florida who worked with pregnant women, and a female researcher at Yale who worked with women and reproductive rights in North and South America.

Interviews were conducted by the first author between October 2016 and June 2017 in English, Brazilian Portuguese, and Spanish. Eleven face-to-face interviews were conducted and 19 remote interviews were conducted by Skype. The average length of interviews was two hours. All authors participated in the development of the interview guide and thematic analysis of interviews. A pilot test of the guide led to small changes in open-ended questions and prompts. The interviews were transcribed verbatim in English, Portuguese, or Spanish by research assistants who were native speakers of each language. The first, second and third authors also transcribed the interviews. Dr. Linde’s native language is Spanish, but she is also fluent in Portuguese and English. Dr. Tristan-Cheever’s native languages are Spanish and Portuguese, but she is also fluent in English. Ms. Furtado is Puerto Rican and fluent in English and Spanish. The audiotaped and transcribed interviews were kept anonymous. Dr. Linde double-checked all recordings for accuracy.

### 2.2. Data Analysis

The data transcripts were analyzed using thematic analysis, informed by a phenomenological perspective in order to identify and analyze patterns and themes in the qualitative data [18]. The thematic analysis was approached as a ‘bottom-up’ process in which all data in the transcripts were examined and analyzed for patterns of meaning to explore what themes were important in addressing the research questions. Transcripts were systematically coded by Dr. Linde using NVivo software^®^ (QSR International Americas Inc., MA, USA) for data analysis. An inductive analysis was performed rather than trying to fit the codes into a pre-existing coding frame [19]. The process began by generating a few free nodes; then, ideas or key words derived from the interviews were used to code the text into themes. Dr. Linde collaborated with Dr. Siqueira and Dr. Tristan-Cheever to create the structure of the node tree. Dr. Siqueira and Dr. Tristan-Cheever coded several interviews independently and discussed the node tree with Dr. Linde in several meetings before consensus was achieved. We adopted pseudonyms for all participants to maintain anonymity.

### 2.3. Ethical Considerations

The research protocol was approved by the Institutional Review Board of the University of Massachusetts, Boston, under number 2016186. All participants were informed of the study aim and procedures and advised that participation was voluntary and confidential. Written consent was obtained from those who agreed to participate in the study.

## 3. Results

### 3.1. Participant Characteristics

Table 1 displays the sociodemographic characteristics of the participants. The ages of the women varied between 22 and 41 years of age. Twelve women had long-term partners, while 18 were married. The women resided in Florida, Massachusetts, Washington D.C., Puerto Rico and several localities in Brazil. Participants self-reported their ethnicities as Brazilian, Hispanic, and American. Sixteen participants had been pregnant recently or were pregnant at the time of the interview, while eight planned to become pregnant and six did not want to become pregnant but lived in locations affected by the Zika virus. Two participants were misdiagnosed with Zika infection while pregnant, two had a husband diagnosed with the virus while pregnant, one was suspected of bearing a child with microcephaly, and six had a positive diagnosis of Zika though not pregnant. All participants had at least high school education. Four held PhD degrees, three held master’s degrees, three had completed postgraduate studies, and seven had college degrees. Participant religious beliefs were the following: 18 Catholic, five Evangelicals, five Spiritist, one Agnostic, and one Atheist.

### 3.2. Thematic Analysis

Our analysis identified several overarching themes related to the impact of Zika on women’s family and personal lives. The themes elicited from interviews with lay persons and professionals were quite similar. Therefore, we integrated their answers in the same data set. Table 2 presents an overview of the themes, categories, and subcategories.

#### 3.2.1. Biased Pressures on Women: “It Is Too Much to Ask, Too Much to Handle”

Many participants reported that there were social pressures on them to make sure that they did not contract the Zika virus. Many women felt that the government recommended measures inadequate for truly avoiding infection, while the accountability for avoiding the infection was placed solely on them. The recommended measures focused on changing daily habits, such as avoiding geographic locations likely to have intense infestations of the Aedes mosquitoes, covering oneself completely with clothing, and applying repellents that were persistent enough to stay on the body for extended periods. The pressure to avoid contagion led many women, facing the potential to be infected with Zika, to feel guilty and lonely. As Interviewee 1 summed up:

“*I felt I had failed at doing what I had especially and necessarily to do… and it’s like… okay you are telling women who live in a tropical climate not to get mosquito bites. It is like basically saying that if you do [get Zika], it is your fault*.”

The general perception was that women were given the responsibility of staying healthy and Zika free, which placed an undue burden on them, as Interviewee 2 from Brazil, blatantly asked:

“*So, if you have a child with microcephaly who is to blame?*”

Some women complained that government recommendations intruded into their personal lives. The public health establishment advised women to avoid sexual intimacy with their partners to circumvent an unexpected pregnancy that could lead to complications resulting from Zika virus infection. The recommendations to use chemical repellents and stay away from mosquito-infested locations created an enormous pressure on women to change their daily routines. Participants mentioned that those recommendations only targeted women and interpreted them as an unspoken gender bias, because all the burden of personal protection against mosquito bites was placed on women. According to Interviewee 3, the Brazilian government was not accountable for preventing or eradicating Zika:

“*I think, when the Minister [of Health] stated that women should not become pregnant, I found [it to be] an invasion. I think that he should be concerned with public policies that solve the [Zika] problem and not with recommendations about when women [should] get pregnant*.”

Others, like Interviewee 4, perceived that the advice was relevant, but overburdened the individual:

“*I agree that they should inform people [about Zika], but at the same time I think that to ask to not have sexual relations, it is too much to ask.*”

There are a number of factors that influence whether any person—men or women—may become infected with any type of mosquito-borne illness, but as suggested by interviewees, there was significant silent suffering by women facing the threat of exposure to the Zika virus. As Key Informant 1, from Brazil, explained:

“*For any woman who lives in a place where Zika is part of everyday life, it is terrifying*.”

#### 3.2.2. Fear of the Unknown: “So I Had the Feeling of Not Knowing What It Was”

All participants stated that they experienced feelings of overall uncertainty that manifested as fear, insecurity, worry, and stress. Many of these feelings resulted from lack of knowledge about the Zika virus. This fear was prevalent among pregnant and non-pregnant women, those in the midst of planning to have children, as well as among women who lived in places where infected mosquitoes were present. The women interviewed stated that Zika was an unknown epidemic that caused innumerable questions to surface, such as how the virus could be contracted; whether or not they would be infected by the virus; the medical impacts of Zika; and whether treatment would be available if they were infected. This scientific uncertainty about the virus increased their feelings of apprehension due to their perceived inability to properly protect themselves against mosquito bites. Interviewee 5, from Miami, explained the anxiety Zika created:

“*It is such stress, you are all the time worried if there is a mosquito around you, and it is a constant focus of tension… you live in permanent tension and angst.*”

The lack of medical knowledge about Zika also heightened feelings of insecurity. Some women did not trust the medical community’s capability for tackling Zika. Those women were skeptical of the medical community’s true knowledge of the virus, given the lack of agreement among medical practitioners. They were concerned with the suggested forms of combatting the mosquito through continuous application of chemical repellents and doubted the alleged long-term health effects after infection. Several women perceived a lack of interest from their doctors regarding their concerns, which created a cycle of worry and stress, stemming from having a medical provider who was not adequately prepared to deal with the illness. 

Key informant 2 recounted the stress encountered when facing the ignorance of a physician who was not fully informed about how to diagnose and treat Zika:

“*I was more worried, definitely more worried because if the doctor, who was a doctor, didn’t know what was going on, how was I, being just me, gonna feel right… “[the doctor said] ‘I don’t know if it is Zika, because I never treated Zika*.”

The education levels of participants greatly influenced their attitudes towards Zika. Those interviewed with academic training or higher levels of education had a tendency to be consumed with high levels of worry and stress about the epidemic. Those women perceived the limitations in the educational information provided to the public, the inadequate government actions towards improving public health, and the inadequate medical care provided. Quite the opposite impact occurred among women with less education who accepted, without questions, information from medical providers or other health-related organizations and exhibited a calmer outlook about their predicament. These two opposing perceptions can be surmised from the distinct outlooks of Interviewee 6, from Miami, and Interviewee 8, from Puerto Rico:

“*Well yeah, unfortunately, I know necessarily more than I ever wanted to know or thought that I would have to know in my life*.”

“*The information that has circulated here is that the first three months are crucial for the baby if it does not come with microcephaly. That’s the information that they gave us here and it satisfies me*.”

#### 3.2.3. Lifestyle Changes: “I Had Made My Life So Miserable Not to Have This [Contract Zika] Happen.”

The daily lives of the participants interviewed, as reflected in their social relationships and interactions with their partners, family members and children, were greatly impacted by Zika. Women described encountering obstacles in providing attention and care to children because they were preoccupied with their health, particularly if they were pregnant or attempting to conceive. They revealed that intimate relationships with partners suffered both emotionally and sexually, as the fear that Zika could be contracted via sexual contact caused a strain in the partnership. They showed resignation with respect to familial relationships by branching out of the nuclear family. Despite residing in the same city of their kinfolk, they would not visit them often, suggesting poor interpersonal interactions due to feelings of isolation and seclusion that resulted from fear of Zika. Interviewee 9, for example, detailed her personal experience:

“…It affected [the relationship] because [Zika] is a stress. You are worried, all of the time if there is or isn’t a mosquito [present]… a constant focus of tension, the quality of life falls a lot because that affects the relationship. I had places that I would not go to… because I thought there could be mosquitoes.”

Women, in order to protect themselves from the threat of the virus, adopted behaviors that caused substantial changes in their social lives, identities, and personal wellbeing. These new behaviors caused deep disruptions in their psychosocial status. They often sprayed themselves with chemical repellents throughout the day and almost always wore long dresses and long sleeve shirts, despite residing in tropical climates with warm temperatures. One participant even relocated from Florida to Canada. A significant adjustment of what should be considered normal routine behavior was described by Interviewee 9:

“[I] bought a repellent and developed a routine that I put on every time I showered. I used to put on as if it were cream… like brushing your teeth and I would put it on every time I went outside. I tried to put on long clothes and not open shoes. I bought a product that I never used, to put on [my] clothes. [It] was very strong because, at the same time, these are chemicals to protect against Zika, but I’m also pregnant with a lot of chemicals all day long.”

On a personal level, participants described Zika as taking away the enjoyment of being a woman, especially when referencing motherhood and femininity. As Interviewee 10 stated,

“*I had become pregnant. This was a very happy moment. I wanted to show-off my belly. I wanted to wear dresses [but] I had to put aside my femininity because I had to protect myself*.”

Many women expressed living in constant fear or anxiety of having to avoid or prevent mosquito bites from affecting their own health or that of their unborn child. A participant actually mentioned that she was not going to have any other kids after the epidemic and she didn’t really get to enjoy her pregnancy.

Furthermore, some women completely rejected embarking on the journey of motherhood altogether, because becoming pregnant was a source of worry, pain, or stress. The concept of femininity also spilled over into the emotional and sexual relationships women had with their intimate partners. By reflecting on the external relationships that can be affected by Zika, participants gave us insight regarding how the virus shrouded their internal sense of self. The fact that participants felt the need to take drastic measures to avoid becoming infected subdued positive emotions surrounding pregnancy, motherhood, and femininity.

## 4. Discussion 

Few studies in the literature address the experiences of women indirectly affected and less vulnerable to the effects of the Zika epidemic [20]. Most research published so far has primarily focused on women with children affected by Zika and/or living in areas where the Zika virus was widespread [21,22,23]. Those studies showed that the repercussions of Zika were a huge burden on already vulnerable women that already lived in precarious social conditions and those conditions were exacerbated further by it [23]. The discussion about infected women has concentrated on strategies to limit or prevent reproduction (i.e., abortion) in order to reduce the dissemination of the Zika virus [24,25,26]. Additionally, studies mainly address the attitudes, knowledge, and behaviors of pregnant women [22,24,27]. In our study, women lived in diverse geographical regions and were from different nationalities and ethnicities, but similar socioeconomic backgrounds. Our study demonstrates how the Zika epidemic affects all women to some extent. It shows that the social effects of the epidemic affect more women than had been thought before and at deeper and psychosocial levels. 

The framing of epidemics defines public health policies and health research [28,29]. Some studies that showed how Zika’s representation as an epidemic centered on mosquitoes and women are based on a limited conceptualization of virus transmission, human sexuality and reproduction [30]. The Zika epidemic showed once more that public health policies to control epidemics produced narratives suggesting that women were responsible for controlling the virus, since their bodies were the most affected. On the other hand, they were the most neglected by this limited framework. A number of women in our study perceived that the public health systems placed an unfair responsibility for preventing health complications from Zika onto women who had limited ability to eradicate the vector. Many women stated that those measures were invasive, while creating the perception that they were the sole determinant of whether or not they contracted Zika. Their perception complements the findings of studies that identified two main frameworks relied upon by public health officials and the media to control the Zika epidemic: one focused on eradicating the vector (mosquito) and another on preventing microcephaly, both of which place the burden of prevention on women [31].

It has been claimed that Zika is a complex issue because women are not asked to protect their own health, but that of a child [32]. However, the Zika epidemic seems to be quite similar to other cases of global epidemics. For example, the policy and social responses to Zika were similar to the ones seen in the case of German measles or Rubella outbreaks, when the priority was also to protect future fetuses rather than women’s bodies [33]. Because of the focus on preserving the life of the fetus, women had to deal with particular public health questions and costs [34]. The Rubella outbreaks also have aspects in common with the Zika outbreak, such as the gender bias in making women responsible for most personal protective measures [35,36]. Thus, women appear to be disproportionately affected by epidemic outbreaks that have reproductive implications, even when they are only indirectly affected. Based on past experiences and our data, we argue that gender inequality and gender discrimination must be recognized and rendered visible in the public health emergency response against epidemics.

Lack of scientific evidence, such as the physiopathology of Zika and effective protective measures against the virus, caused a variety of questions to emerge during the early days of the epidemic. The lack of public and medical knowledge about Zika contributed to uncertainty and insecurity. A similar phenomenon was evident during the 2009–2010 influenza pandemic, when individuals experienced confusion, anxiety, and increased risky behaviors because of uncertainties [37]. Our findings suggest that the Zika epidemic led to similar anxiety among women who might not be directly impacted by Zika, but still experienced high levels of apprehension. The lack of knowledge among medical professionals caused fear and anxiety to participants, coupled with public health communications that made individuals responsible for the spread of the virus because of lack of knowledge of the virus and inadequate social policies to control its transmission.

A number of studies reported that women who had lower education attainment levels were significantly less knowledgeable about the information produced for public consumption [27,38]. Participants with higher levels of education had ample access to reliable sources of information and demonstrated high levels of scientific knowledge. Our study suggests that women with higher levels of education understood the limitations of the information, government actions, and medical care they received, which ended up producing higher levels of anguish and worry. The opposite seemed to be the case for women with lower levels of education.

It was previously reported that women with children affected by Zika showed a significant loss of a sense of themselves [39]. A 2016 study that focused on pregnant women impacted by Zika found that women in Brazil and Puerto Rico, infected and not infected with the Zika virus, had high levels of stress, anxiety, and depression [40,41]. Our study adds to the literature by suggesting that even women who were not pregnant and only indirectly affected by Zika made decisions and arrangements to prevent infection that caused significant changes and disruptions to the very essence of their being.

Epidemic outbreaks, such as Ebola, create long-term distress in members of families of those who succumbed to the disease. Individuals experienced fear, anxiousness, numbness, and detachment [37]. Whole communities experienced fear, isolation, and suffering during or after an infectious disease epidemic [42,43]. In our case, the women interviewed expressed, both directly and indirectly, suffering at the individual and family levels. The changes in their lives were significant, and to the best of our knowledge, we report it for the first time in relationship to the Zika epidemic.

Another profound impact of the Zika epidemic on the lives of many Latina and Brazilian women interviewed was the loss of femininity, because they had to cover their bodies to protect themselves against mosquito bites and could not dress as normal Brazilian or Latina women do. For those interviewees, their body was an important part of their femininity [44,45,46] and having to cover themselves prevented their ability to freely display their womanhood. They associated a sense of empowerment with the ability to exhibit one’s body and/or pregnant body.

Our study is limited by a small convenient sample, which may be biased for not including a diverse representation of women due to the sampling method. However, it is conceptually generalizable in the sense that the themes that emerged are relevant to analyze and guide the response by women indirectly affected by Zika. Additionally, we may not have comprehensively analyzed the effects of Zika on women’s health due to our expertise, which is limited to public health and biological sciences. 

However, through one-on-one interviews, we developed an in-depth understanding of the Zika epidemic impacts on women’s daily lives. Our analysis of the impacts of Zika on a small sample of women provides input for larger survey studies. Further research may compare the experiences of women directly and indirectly affected by Zika in countries affected by the epidemic. 

## 5. Conclusions

The Zika epidemic highlights the fact that on a global scale, women’s health continues to encounter numerous barriers. Though participants in this study were of different nationalities and ethnicities, and most had good education levels, their actions and thoughts were embedded in common sociocultural norms. Therefore, our study shows the importance of considering cultural aspects and behaviors when implementing health prevention or protection measures to control epidemics. Financial, social, religious and cultural aspects are always involved in epidemics. The Zika epidemic was yet another lost opportunity for the improvement of women’s health by strengthening culturally sensitive family planning services, as the unique costs of the mosquito-borne virus for maternal and perinatal health called for a broad spectrum of public health interventions.

## Figures and Tables

**Table 1:** Sociodemographic characteristics of the women interviewed.

Characteristic	N
N	30
Age Range	
22–30	12
31–41	18
Self-Reported Ethnicity	
Brazilian	13
Hispanic	12
American	5
Civil Status	
Married	18
Single/In a Relationship	12
Maternity status	
Recently born baby	6
Pregnant	8
Planning to get pregnant	6
No plan to get pregnant	4
Residence	
Brazil	10
Washington D.C.	1
Massachusetts	5
Florida	7
Puerto Rico	7

**Table 2:** Summary of themes and sub-themes.

Themes	Sub-Themes
Perceptions of public policies	Acceptance and trust Discontent Skepticism Conflicting feelings
Information and health literacy	Formal/official channels Informal channels Rumors vs facts False information Attitude Awareness of limitations Trust Intimidation
Perceptions of medical care	Positive attitude and trust Awareness of the lack of knowledge Conflict and contradiction
Impacts on daily life	Changes in routines Impacts on professional life Impacts on family Drastic changes Impacts on mental wellbeing
